# Improving the Early Age Strength of Eco-Efficient Mortar with Low Clinker Content Considering Binder Granulometry and Chemical Additives

**DOI:** 10.3390/ma17184509

**Published:** 2024-09-13

**Authors:** Tobias Schack, Bastian Strybny, Michael Haist

**Affiliations:** Institute of Building Materials Science, Leibniz University Hannover, 30167 Hanover, Germany; b.strybny@baustoff.uni-hannover.de (B.S.); haist@baustoff.uni-hannover.de (M.H.)

**Keywords:** eco-efficient mortar, early age strength, C-S-H seeding, slag fineness, clinker content

## Abstract

In this study, the impact of binder granulometry as well as chemical additives on the early strength and early age stiffness development of eco-efficient mortars with low clinker content and ternary blended cements with high contents of slag and limestone powder was investigated. With regard to granulometry, the particle size distribution of the slag was varied in two steps. In addition, admixtures based on nano-granular C-S-H seeds were used for the acceleration of the hydration reaction. Both the compressive strength at 1d and the in-situ ultrasound pulse velocity for the first 36 h were determined. The granulometric optimization of the slag leads to an improvement in compressive strength of up to 162% in the early phase of the first 24 h. In addition, C-S-H seeding enhanced the hydration reaction mortars as early as 6 h after water addition (at 20 °C). An increase in the dosage level of the C-S-H seeding admixture further resulted in a corresponding increase in early strength, particularly when the clinker content exceeds 30 wt% up to 103%. The proposed combined granulometric and admixture-based approach significantly improves the reaction of the binder in the early phase, so that the clinker factor can be significantly reduced while maintaining a comparable binder intensity.

## 1. Introduction

The cement and concrete industry have introduced various measures to reduce CO_2_ emissions in cement production, including a.o. energy efficiency, the use of alternative fuels and replacing Portland cement clinker with supplementary cementitious materials (SCM) [1,2,3]. 

However, the individual options differ greatly in terms of the technical measures and results as well as the normative requirements and the necessary investments. On the specific technological side, there are essentially two possible paths to CO_2_-reduction:

1. Application of multi-component cements (CEM II to CEM VI) containing significant amounts of supplementary cementitious materials to replace Portland cement clinker [3,4,5].
2. Use of new clinker formulations, e.g., based on calcium sulfoaluminate [6] or belite [7].

The large majority of cements and concretes produced in Europe relies on approach (1), using SCMs as clinker substitutes to reduce the environmental footprint [8]. In doing so, substantial experience exists in guaranteeing equal mechanical performance and sufficient durability at the design age, especially for clinker contents in the range as low as 50 wt% and eventually even lower e.g., looking at high slag cements [9,10]. However, looking to the early stage of hydration, the replacement of Portland cement clinker with SCMs generally goes along with a substantial reduction of strength as well as other mechanical properties, which is due to the slower hydration kinetics of SCMs [11]. In construction practice, e.g., for precast elements, early age strength however is a main factor in enabling early demolding and accelerating the construction process, this being essential for an economical production. Guaranteeing early age strength is therefore a key factor, which currently severely limits clinker substitution in today’s precast concrete industry [12].

In order to increase early strength despite low clinker content, various countermeasures are available, e.g., increasing the temperature of the concrete during curing [13], increasing the grinding fineness and thus the surface area of the reactive components as to accelerate the hydration reaction [14] or enhancing C-S-H nucleation by the addition of finely ground limestone powder [15,16] or Calcium-Silicate-Hydrate (C-S-H) seeds [17]. In precast elements production even for pure Portland Cement (OPC)-based systems, increasing the cement fineness is standard practice e.g., by using a CEM I 52.5 R with high specific surface areas and many plants, heating chambers are also employed despite high energy costs. As the heat curing temperature however is limited (e.g., [18,19]), in order to prevent secondary ettringite formation [20], the potential of a heat curing approach to bridge the early age strength gap between pure OPC concretes and concretes with low clinker content is seen as negligible.

In contrast, increasing the degree of fineness through grinding the reactive binder components offers a very large potential [14,21,22,23,24,25]. Particularly in the case of ternary blended cements, e.g., OPC-slag-limestone cements; however, the manifold interactions of the individual cement components must be taken into account [26]. In [27] it was shown that the binder components fineness significantly influences the hydration kinetics and the resulting mechanical properties. The development of early age strength is significantly influenced by the cement clinker fineness, and here especially due to the ultra-fine particles (d_90_ < 8.0 μm) [28]. A higher slag fineness has a significant effect on hydration after two days and at a later age [29]. Zhou and Zhang [22] studied the hydration of ultrafine slag (d_50_ < 2.0 μm) and concluded that the hydration of ultra-fine slag is 67% faster compared to normal slag (d_50_ < 10.0 μm). As the fineness of the slag increases, the phase assemblage changes and more performance efficient microstructures with a larger volume of C-S-H phases are formed [27]. As a result of the higher fineness of the cement constituents, the clinker can be replaced by less reactive materials. For example, a very low OPC clinker content of approx. 30 wt% and a high proportion of approx. 40 wt% limestone powder with a w/b ratio of 0.45 can be used in principle for structural concrete when using fine slag [30]. In addition to enhanced mechanical properties, these concretes also exhibit improved durability properties. It should however be noted that both the energy demand and the costs for the grinding process significantly increase with increasing fineness of the materials [25]. Grinding clinker requires less energy than grinding slag [27]. This increased energy requirement may have a negative impact on efforts to reduce the carbon footprint of cement production and will definitely have an impact on economic viability. Therefore, the economics, performance and environmental impact of such highly substituted cements must be considered together.

A different approach for increasing the early age strength of concrete lies in the use of chemical admixtures. Several studies in the literature demonstrated that C-S-H seeding can significantly improve the hydration and strength at an early stage of OPC [31,32,33,34] and of blended cements [35,36,37,38]. C-S-H seeds boosts the formation of new hydration products by strongly enhancing C-S-H nucleation and precipitation. As C-S-H is the primary strength determining hydration product in concrete [39], acceleration C-S-H formation also enhances strength formation. In particular, C-S-H seeds accelerate the hydration of Tricalcium Silicate (C_3_S) which can be accelerated by using C-S-H seeds. Alizadeh et al. [40] showed that synthetic C-S-H seeds accelerate the dissolution of C_3_S and the nucleation of C-S-H. In addition, Thomas et al. [41] showed that relatively small additions of C-S-H seeds to OPC or pure C_3_S paste lead to an elimination of the induction period and the main hydration peak occurs earlier. A comprehensive overview of the principles and application of C-S-H seeds to accelerate hydration can be found in [17].

As described above for the production of precast elements, early age strength is a key factor in enabling early demolding and accelerating the construction process. This currently severely limits the substitution of clinker in today’s precast concrete industry. Therefore, the focus of the investigation in the paper at hand was on the interactions between the granulometry of the binders and the use of chemical additives and the resulting influences on early age strength. For this purpose, mortar samples were produced instead of concrete to facilitate the procedure and to allow for a broader investigation matrix. However, the findings are easily transferable to concrete. The clinker content in the binder mixes was gradually reduced and compensated by increasing the proportion of limestone powder. The slag content remained constant, reflecting the limited availability of this component. On all samples, the mechanical properties and the microstructural development were examined. In addition, the mortars are evaluated in terms of sustainability and ecological performance. The results show that both granulometric approaches as well as admixtures are suitable pathways for the design of eco-efficient mortars with low clinker content and high early strength. Ternary blended cements with high contents of granulated blast furnace slag and limestone powder can also be used for the production of precast concrete elements, which leads to a significant reduction in the resulting CO_2_ emissions.

## 2. Materials and Methods

### 2.1. Raw Materials and Mortars

An Ordinary Portland Cement CEM I 52.5 R according to EN 197-1 [42] (clinker content ≥ 95 wt%) was used as a reference for the investigations. The sulphate carrier and other minor constituents (up to 5% by weight) were neglected in the following calculation and attributed to the Portland cement clinker. In a next step, ternary blended binders were prepared with different quantities of the previously mentioned OPC (C), limestone powder (LL) and ground granulated blast-furnace slag (S; referred to as slag). Three slag samples (S-R, S-F and S-X) with different degrees of fineness (varying d_50_ values) and a limestone powder (LL) were used to design the blended mixtures in the laboratory. Accordingly, the particle size distribution of the slag was varied in two steps to study its effect on early age strength.

**Figure 1:**
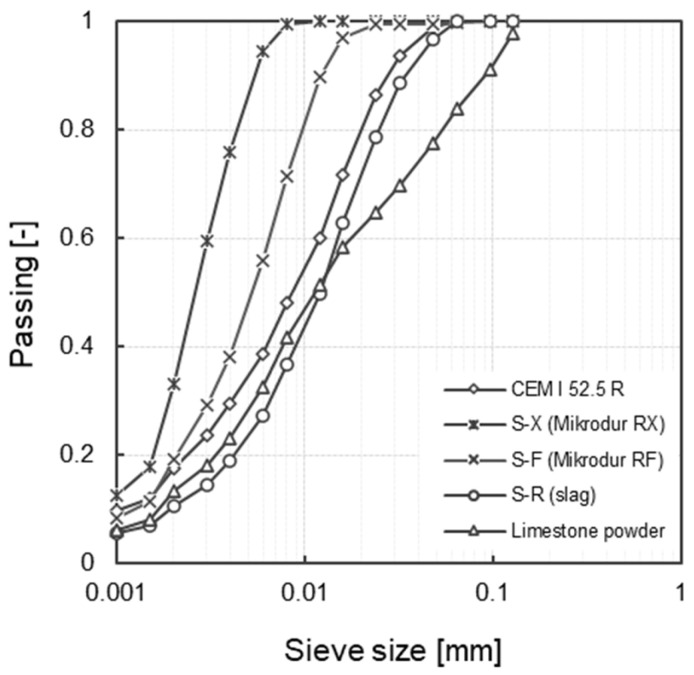
Particle size distribution of the binder components.

The S-R slag was a standardized granulated blast furnace slag (d_50_ = 12.1 μm). The finer variants were MIKRODUR R-F (d_50_ = 4.7 μm) and MIKRODUR R-X (d_50_ = 2.0 μm) from Dyckerhoff GmbH, Wiesbaden, Germany [43]. MIKRODUR R products are ultra-fine binders based on granulated blast furnace slag. The composition corresponds to that of a CEM III/C according to DIN EN 197-1 [42]. In the production process of MIKRODUR, after separate grinding of the raw materials and separate screening to the desired fineness, homogenization is carried out using gypsum as a set agent. The low proportion of clinker and minor constituents of the products was taken into account in the mix design and added to the clinker content of the concrete. The content of the reference cement CEM I 52.5 R was adjusted in each concrete composition. It should be noted that, depending on the clinker content, a small amount of very fine clinker was also included in the concrete compositions (variants with S-F and S-X). In this study, an average clinker content of 12 wt.% according to DIN EN 197-1 [42] (CEM III/C = 5 to 19 wt.% clinker) was assumed in MIKRODUR R-F and MIKRODUR R-X. The physical properties as well as the oxide composition (determined by X-ray fluorescence (XRF); M4 Tornado, Bruker Nano GmbH, Berlin, Germany), the loss of ignition (LOI) according to DIN EN 196-2 [44] of the reference cement, the slag samples and the limestone powder are summarized in Table 1 and Table 2. The grain size distributions of the binder components are shown in Figure 1 (determined with measuring device CILAS Granulometer 715, CILAS, Burladingen, Germany).

Table 3 summarizes the compositions of the mortars investigated in this study. All compositions consisted of the following parts: binder; water; and fine aggregate = 1.0, 0.45, and 2.90. The composition of the binder was systematically varied in the components C, S and LL. The slag content remained constant at 30 wt% and the ratio of OPC to limestone powder was adjusted in individual steps from 30 wt% to 50 wt%. Some of the blended mixtures presented do not comply with current standards such as DIN EN 197-5 [46] (e.g., CEM VI). For mortar compositions with MIKRODUR R-F (S-F) and MIKRODUR R-X (S-X), the cement content (CEM I 52.5 R) was reduced following the assumed clinker content in MIKRODUR R-F and MIKRODUR R-X.

The workability of the mortars was adjusted by adding a constant content of polycarboxylate-based superplasticizer (Master Suna SBS 6080; Master Builders Solutions Deutschland GmbH, Trostberg, Germany). In addition, commercially available C-S-H seeds (Master X-SEED; Master Builders Solutions Deutschland GmbH) were used in various dosage quantities for possible chemical activation in the early stage. Master X-SEED 100 consists of a concentrated suspension of C-S-H nanoparticles [47]. The dosage varied between 1.5 and 5.0 wt% of cement. All mortars were prepared in batches of 1.5 dm^3^ and mixed with a standard mortar mixer. The mixing protocol is shown in Table 4.

### 2.2. Methods

The workability of the mortars was determined by the flow table test according to DIN EN 1015-3 [48]. After filling and raising the cone, fifteen shocks were applied before measuring the flow diameter of the fresh mortar. In addition, the fresh mortar density according to DIN EN 1015-6 [49] and fresh mortar temperature were determined.

The time-dependent ultrasonic pulse velocity (UPV) was monitored on hardening samples of the binder paste with the identical w/b-value as for the mortars. No fine aggregate was added, as this does not influence the early hydration process. In this study, the IP8 ultrasound system (UltraTest GmbH, Achim, Germany) was used as the device for the measurements. The UPVs were measured continuously for 24 h at an interval of 60 s at a constant ambient temperature of 20 ± 2 °C. The distance between the receiver and the transmitter of the measuring forms was 40 mm.

Fresh mortars were cast in prismatic molds of 40 × 40 × 160 mm^3^ and stored in the formwork for 24 h at 20 ± 2 °C. Afterwards, the specimens were demolded and stored under water at 20 ± 2 °C until the time of testing. The compressive strength was determined according to DIN EN 196-1 [50] after 1, 2, 7, 28 and 56 days. Six samples were measured in each case. The data given here are the average values of all corresponding measurements.

In the paper at hand, the change in strength compared to a reference mortar was calculated using the strength values of the sample (f_sample,i_) compared to the reference sample (f_ref._) following Equation (1).

r_act,i_ = (f_sample,i_ − f_ref._)/f_ref._ (1)

In the case of chemical activation by C-S-H seeding, r_act,C-S-H_ was calculated from the values of the samples with C-S-H seeding (f_sample,C-S-H_) in relation to the reference sample without C-S-H seeding (f_ref._).

## 3. Results and Discussion

### 3.1. Early Age Stiffness Development

To evaluate the effects of both slag granulometry and C-S-H seeding on hydration processes and microstructural development at an early age of less than 36 h, in-situ ultrasound pulse measurements were carried out on cement paste. The evolution of ultrasonic pulse velocity reflects the microstructural evolution, as low velocities reflect liquid media, while high velocities are measured for solid media in which connectivities between solid phases develop [51]. The temporal evolvement of the ultrasonic velocity of cement pastes or mortar follows three main stages [51]: The induction period is the first stage for cement paste. The second phase is characterized by a rapid increase in velocities due to the formation of new hydration products. Here, the cement paste develops from a liquid suspension into a solid network. In the third stage, a slower increase in velocities can be observed before an almost constant level is reached. The development of the individual phases depends on many factors, such as the composition [33,52] or the temperature [53]. In this study, the second phase is decisive and is discussed below.

#### 3.1.1. Effect of Slag Fineness

**Figure 2:**
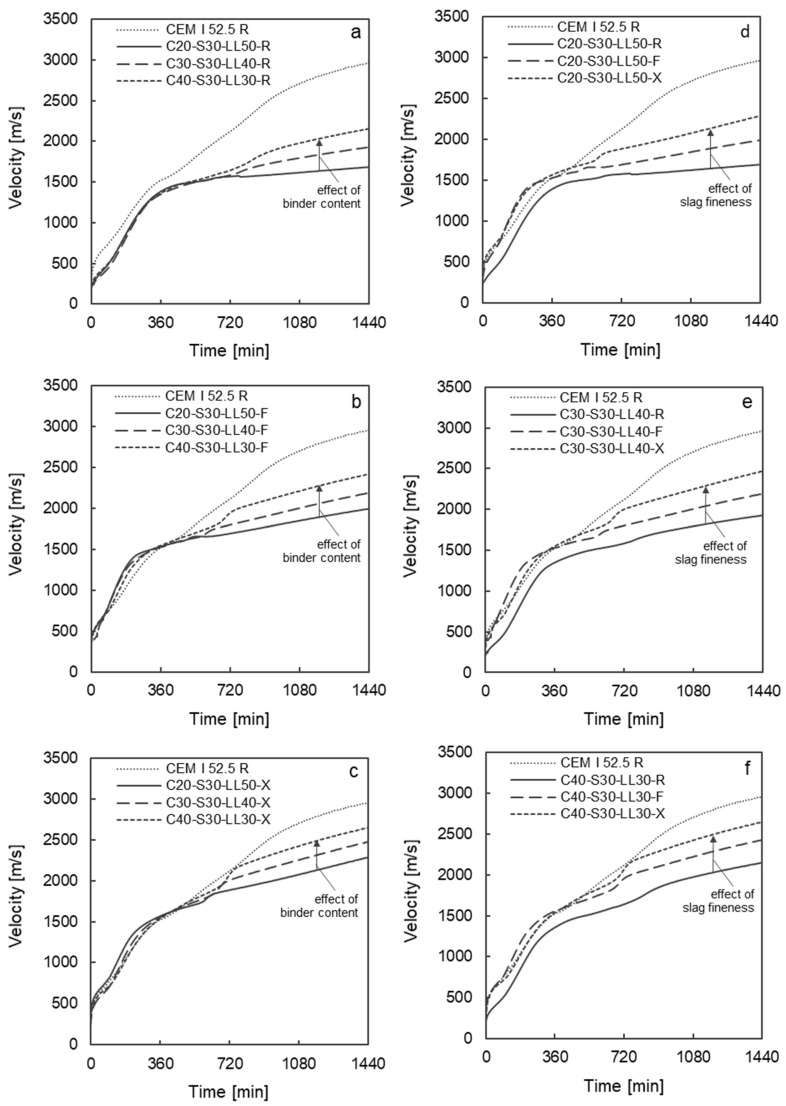
Ultrasonic pulse velocity v evolutions during the first 24 h depending on binder component fineness (S-R, S-F, S-X) and clinker content of 20 wt%, 30 wt% and 40 wt%—left (**a**–**c**): variation of the clinker content with constant slag fineness; right (**d**–**f**): variation of the slag fineness with constant clinker content.

Figure 2a–c shows the UPV curves over time as a function of the clinker content for all investigated slag fineness grades. The influence of the clinker content can be clearly distinguished. In the early phase up to approx. 420 min, the UPV curves are nearly the same for all binders regardless of the clinker content and slag fineness. Only the OPC clinker seams to contribute to the hydration reactions and thus to phase formation in this very early stage. This is also confirmed by the comparison of the UPV curves of the multi-component mixes with the UPV curve of pure CEM I 52.5, which is very similar in this early phase. After approx. 600 min of hydration, the influence of the clinker content is clearly visible. From this point onwards, an increased clinker content leads to an accelerated UPV, which indicates an accelerated hydration reaction resulting in the formation of a stiffer microstructure. This is also shown by the UPV values after 24 h, which rise significantly with increasing clinker content regardless of the slag fineness (cf. Table 5). As expected, an increased early strength after 24 h can be assumed for all investigated slag variations with increased clinker content.

In Figure 2d–f, the influence of the slag fineness on the development of the UPVs can be clearly observed as early as approx. 700 min. With increasing slag fineness, the UPVs increases significantly from this point onwards, regardless of the clinker content. The increase in UPVs is comparable for all clinker contents investigated. Only the absolute values of the UPVs decrease with decreasing clinker content (cf. Table 5). With a very low clinker content of 20 wt%, no further increase in UPV can be detected from around 420 min when using the coarse slag (S-R). From this point onwards, the UPV curve is almost horizontal. With such a low clinker content, the coarse slag does not yet contribute to the hydration reaction and phase formation at this early stage. As a result of the increase in the fineness of the slags, however, a clear increase in the UPV curves can be observed at this low clinker content (cf. Figure 2d–f).

In summary, it can be stated that an increase in the slag fineness leads to accelerated phase formation in the first 24 h in the eco-efficient binder pastes with low clinker content investigated in this study. Binder pastes with a clinker content of 40 to 30 wt.% clinker show a significant increase in UPVs and thus phase formation in the microstructure due to the higher slag fineness. Even with a very low clinker content of 20 wt%, increased phase formation can be detected on the basis of the higher UPVs due to the finer slags.

#### 3.1.2. Effect of C-S-H Seeding

**Figure 3:**
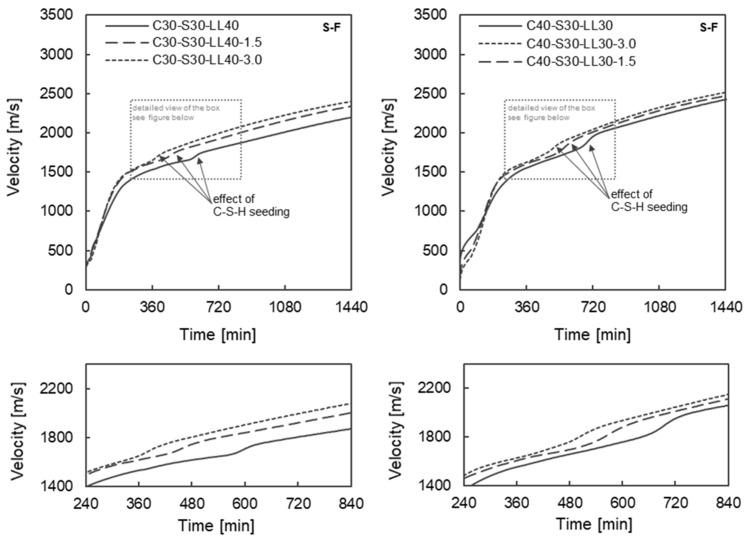
Ultrasonic pulse velocity v evolutions during the first 24 h depending on the C-S-H seeding dosage and clinker content of 30 wt% (**left**) and 40 wt% (**right**)—all pastes shown are with fine slag (S-F).

The effect of the addition of C-S-H seeds on early hydration during the first 24 h was determined on binder pastes with 30 and 40 wt.% clinker and using the fine slag (S-F). The dosage level of C-S-H seeds varied between 1.5 and 4.0 wt% of binder. Figure 3 shows the measured UPVs in the first 24 h as a function of the clinker content (30 wt% = left and 40% = right) and the dosage level of C-S-H seeds.

In the early phase up to approx. 360 min, only the clinker appears to control the hydration reaction. The UPV curves all show comparable values in this period. All binder pastes with 30 wt% clinker show comparable values between 1387 m/s and 1400 m/s after approx. 180 min regardless of the C-S-H seed dosage. In the following period between approx. 360 to 680 min, however, the influence of the C-S-H seeds clearly manifests itself (see Figure 3, bottom row). Without the addition of C-S-H seeds, a steep increase in UPVs can be observed after around 600 min as a result of the additional reaction of the fine slag (cf. Section 3.2.1). This steep increase in UPVs shifts towards an earlier point with increasing C-S-H seed dosage. Thus, a greater difference between the UPVs as a function of the C-S-H dosage can already be determined after approx. 360 min (cf. Table 6). The addition of the C-S-H seeds leads to an accelerated hydration reaction, so that a higher strength is formed at an earlier stage. Consequently, the UPVs increase slightly after 24 h with a higher addition of C-S-H seed, regardless of the clinker content. The UPV increases with a clinker content of 30 wt% (Figure 3, left) from 2254 m/s (without C-S-H seeds) to 2402 m/s by adding 3.0 wt% C-S-H seeds after 1440 min (cf. Table 6). The addition of C-S-H seeds in 30 wt% clinker content mortars therefore appear to lead to an increase in early strength after 24 h. A comparable increase can be observed with a clinker content of 40 wt.% (cf. Table 6), although the absolute values are somewhat higher, as the increased clinker content has led to a faster hydration reaction and increased phase formation during this period.

### 3.2. Compressive Strength

The compressive strength was determined after 1, 2, 7, 28 and 56 days on prismatic specimens according to DIN EN 196-1 [50]. To evaluate the early strength, the 1d-strength is considered below. The strength after 28 days is used to evaluate the resulting strength at an advanced age.

#### 3.2.1. Effect of Slag Fineness

**Figure 4:**
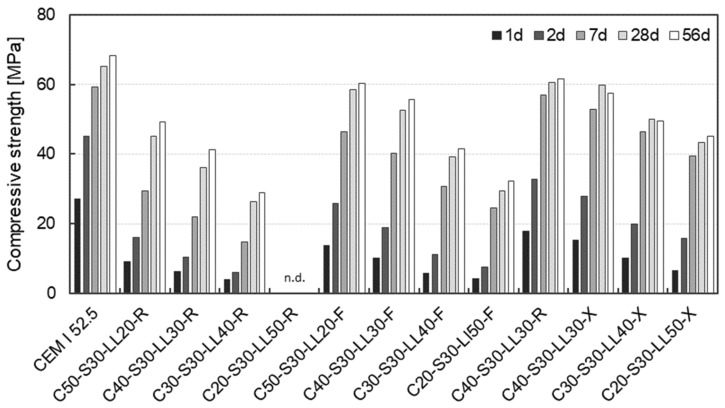
Compressive strength at 1d, 2d, 7d, 28d and 56d, respectively, of all mortars investigated considering the clinker content and the slag fineness (n.d. = not determined).

Figure 4 shows the compressive strength at 1, 2, 7, 28 and 56 days, respectively, for all mortars investigated considering the slag fineness. In general, the results show that the compressive strength increases with increasing clinker content for all ages at the same fineness of slag. When comparing mortars with the same clinker content but different slag fineness, the compressive strength increases with increasing slag fineness (R → F → X) as well. Increasing the slag fineness (R → X) has a comparable effect on the increase in strength as increasing the clinker content e.g., from 20 wt% to 40 wt%. Comparable results were also observed for concretes with these raw materials [30].

**Figure 5:**
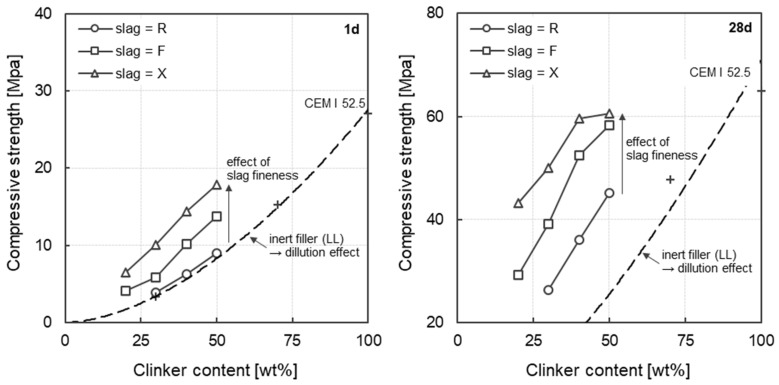
Compressive strength at 1d (**left**) and 28d (**right**) as a function of the clinker content considering the slag fineness.

Figure 5 shows the compressive strength at 1d (left) and 28d (right) as a function of the clinker content from 50 wt% to 20 wt% considering the slag fineness. In addition, the compressive strength of mortars in which the clinker was only replaced with limestone powder (LL) is shown. As limestone powder is inert, the dilution effect and the resulting decrease in compressive strength can be estimated with decreasing clinker content. In terms of slag fineness, it can be observed, that the compressive strength after 28 days (Figure 5, right) increases linearly with increasing clinker content between 20 wt% and 50%, irrespective of the slag fineness. The mortar made with 50 wt% clinker and ultra-fine slag (S-R) exhibited a similar compressive strength of 60.6 MPa as the reference concrete with CEM I 52.5 R of 64.2 MPa at 28 days of age. When using the fine slag (S-F), a comparable compressive strength of 60.1 MPa can be achieved after 28 days with a clinker content of 50 wt%. The slag fineness leads to a quasi-parallel shift of the individual straight lines. With increasing fineness of the slag (R → F → X), higher compressive strengths after 28 days are observed with a constant clinker content. These results show that the granulometric adjustment of the slag enables a significant reduction of the clinker when considering the compressive strength after 28 days. Thus, with increasing fineness of the slag (R → F → X) and a constant clinker content, a denser pore structure and a more efficient microstructure can be assumed. This is also shown by other results from the literature [14,29]. Zhou and Zhang [22] also observed accelerated hydration when using ultra-fine slag (d_50_ < 2.0 μm) compared to normal slag (d_50_ < 10.0 μm). The accelerating hydration, which depends on the slag fineness, can be well observed in the compressive strength after 1 day (cf. Figure 5, left). With increasing fineness of the slag (R → F → X), higher compressive strengths are observed with a constant clinker content. With a clinker content of 50 wt%, the compressive strength increases from 8.1 MPa (S-R) to 18.0 MPa (S-X) due to the increased fineness of the slag. This corresponds to an increase in early strength of 121%. With clinker contents of 40 wt% and 30 wt%, a comparable percentage increase in early strength of 128% and 162%, respectively, can be observed after 1 day. In addition, it can be seen that the coarse slag is not yet involved in the hydration reaction at this early stage, as the corresponding data points nearly coincide with the line shown by pure limestone powder (LL). In these mortars, therefore, only the remaining clinker content leads to a strength-forming reaction. With decreasing clinker content, a decrease in compressive strength can be observed for these mortars (cf. dashed line in Figure 5, left). Based on these results, it can be summarized that the granulometric adjustment of the slag leads to an improvement in compressive strength even in the very early phase. As a conclusion, by increasing the fineness of the slag, the clinker content can be reduced from 50 wt% to 30 wt% without significantly affecting the 1d early strength of 10 MPa.

#### 3.2.2. Effect of C-S-H Seeding

The impact of C-S-H seed dosage on the compressive strength was studied on mortars with the fine (S-F) and ultra-fine slag (S-X) and a clinker content ranging between 20 and 50 wt%. The dosage of the C-S-H seed admixture varied between 1.5 and 5.0 wt%. of binder.

**Figure 6:**
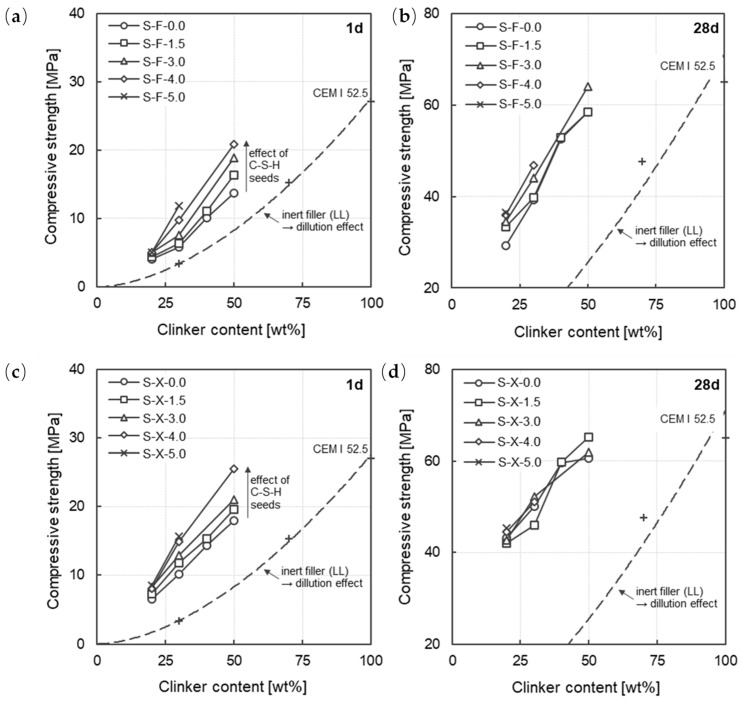
Compressive strength as a function of the clinker content for all mortars investigated (sulfate carrier and other minor constituents (up to 5 wt.%) in the cements were neglected and attributed to the clinker component) considering the slag fineness (S-F = upper row; S-X = bottom row) and C-S-H seed dosage (up to 5.0 wt% of binder)—(**a**) S-F, 1d; (**b**) S-F, 28d; (**c**) S-X, 1d; (**d**) S-X, 28d.

The compressive strength at 28 days is controlled by the clinker content. Regardless of the C-S-H seed dosage level, comparable strengths can be observed after 28 days. The compressive strength increases linearly with increasing clinker content (cf. Figure 6, right). In addition, the increased slag fineness (F → X) leads to increased compressive strength after 28 days. The influence of slag fineness on compressive strength has already been discussed in Section 3.2.1.

**Figure 7:**
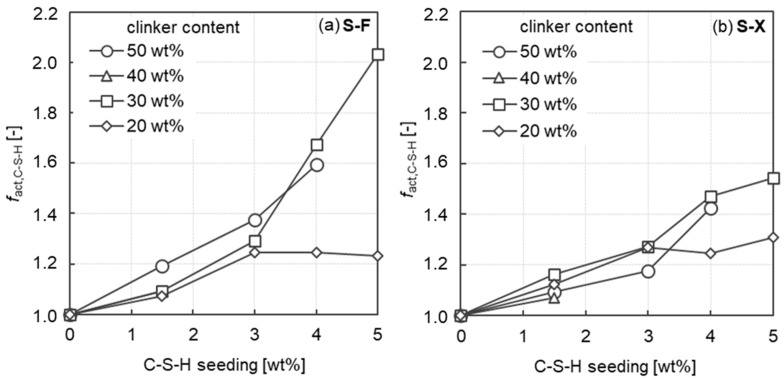
Relative increments in compressive strength after 1 day (r_act,C-S-H_) as a function of the C-S-H seeding of all mortars investigated—(**a**) slag fineness S-F and (**b**) slag fineness S-X.

Compared to the mortar without C-S-H seeding, the compressive strength in the early stage is significantly enhanced with C-S-H seeding particularly for mortars at a clinker content of 50 wt%. The increase in strength for the mortar with 4.0 wt% C-S-H seeding compared to the respective reference mortar without C-S-H seeding are 59% for the fine slag (S-F) and 42% for the ultra-fine slag (S-X) mortar. Comparing these results to mortars with a clinker content of 20 wt% and yet the same C-S-H seeding dosage of 4.0 wt% shows a 24% improvement for the S-F slag and a 25% improvement for the ultra-fine slag, respectively. The relative increments in compressive strength after 1 day (r_act,C-S-H_) as a function of the C-S-H seeding of all mortars investigated is summarized in Figure 7. The values of the relative increments are presented in Table 7. The strength increase as a result of the C-S-H seeding can be clearly observed. As the C-S-H seed dosage level increases, a clear increase in compressive strength can be observed with a clinker content higher than 30 wt%. With a clinker content of 20 wt%, however, the increase in compressive strength stagnates at a C-S-H dosage level of 3.0 wt%. Li et al. [35] found comparable results, whereby the efficiency for increasing the early strength of the C-S-H seeds decreased with increasing substitution of clinker for slag. Further systematic investigations, e.g., on phase development in the first 24 h, are necessary to determine the exact causes. Regardless of the C-S-H dosage higher than 3.0 wt%, an increase in compressive strength of only around 25% can be observed with this low clinker content of 20 wt%. Looking to Figure 7, right, it can be further stated, that with very high slag fineness, the effect of clinker dosage on the C-S-H-seeding effect seems to vanish, with all mortars reacting similarly to the C-S-H seeding.

### 3.3. Sustainability Assessment and Ecological Performance

In order to assess the sustainability of the investigated mixes, the global warming potential (GWP) of the mortar mixes was calculated based on the specific GWP of each raw material and its respective dosage. Here, only the emissions of the life-cycle phases A1 to A3 acc. to DIN EN 15804 [54] were taken into account. The GWP_CO2,e_ was calculated using the following Equation (2).

GWP_CO2,e_ = ∑ n_i_ (p_i,CO2,e_ + g_i,CO2,e_) (2)

Here, n_i_ describes the mass proportion of the raw material in 1 m^3^ of fresh concrete. The variable p_i,CO2,e_ describes the specific CO_2_ emissions associated with the production of the material (see Table 8). Due to the importance of the grinding process, in Equation (2) the specific emissions associated with the grinding g_i,CO2,e_ are considered. The effect of the grinding process was only taken into account for finer grinding of the slag (R → F → X). No reliable data for the grinding processes could be found in the literature, so assumptions were made for the calculation. A detailed description of the calculation of the proportions of the grinding process is given in [29]. Table 8 provides an overview of the global warming potential data of the raw materials used in this assessment.

**Figure 8:**
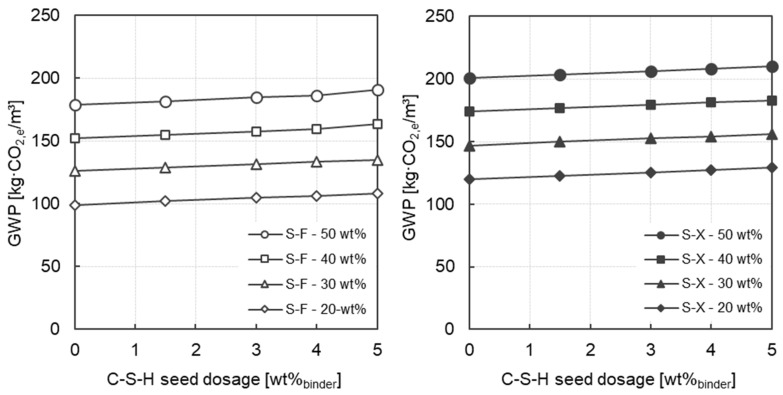
GWP as a function of the C-S-H seeds dosage for all mortars investigated considering the slag fineness (**left**: fine slag (S-F) and **right**: ultra-fine slag (S-X)).

The specific GWP values of all mortars are shown in Figure 8. At 287 kg CO_2,e_/m^3^, the reference concrete with CEM I 52.5 R has a significantly higher ecological impact than the eco-efficient mortars investigated in this study. The GWP values of the mortars substantially decrease with decreasing clinker content. Depending on the clinker content, these mortars have values ranging from 99 kg CO_2,e_/m^3^ to 201 kg CO_2,e_/m^3^ (without C-S-H seeding). With increasing dosage of the C-S-H seeding admixture, the GWP values increase slightly regardless of the slag fineness. Due to the relatively low dosage of C-S-H seeds (up to 5.0 wt%_binder_), this effect is considered negligible compared to the effect of the clinker content. Furthermore, the effect of the increased energy demand required for the grinding process of the granulated blast furnace slag can also be observed in Figure 8 (left → S-F and right → S-X). However, this effect is smaller than the effect of the clinker content. In these calculations, CO_2_ emissions increase by around 12% due to the higher energy demand for grinding the slag (S-F → S-X). In contrast, the effect of the increased clinker content on the resulting CO_2_ emissions is between 16% and 27%. In the presented calculations, the energy related CO_2_ emissions for grinding were calculated using a Germany-specific mean value of 428 g CO_2,e_/kWh depending on the German energy mix (see [58]). However, it should be noted that such grinding could potentially be carried out on the basis of renewable energy, which would significantly reduce the CO_2_ emissions. A reduction in clinker content of around 10% is required to compensate for the increased emissions from grinding the slag.

By calculating the binder intensity [59], the ecological performance can be evaluated, i.e., the amount (kg) of binder to generate 1 MPa of strength at 28 days in 1 m^3^ of concrete. In this study, the binder intensity was converted into the early age binder intensity (b_i,1d_) by using the compressive strength at 1 day instead. This corresponds to the total amount of reactive binder required to achieve a compressive strength of 1 MPa after one day and was calculated using the following Equation (3).

b_i,1d_ = m_binder,reac._/*f*_sample,1d_ (3)

Here, m_binder,reac._ describes the amount of reactive binder in 1 m^3^ mortar and *f*_sample,1d_ describes the compressive strength after one day. Similarly, the early age CO_2_-intensity (c_i,1d_) was calculated using the following Equation (4). The GWP values of the mortars investigated were calculated in relation to the early compressive strength, so that the amount of CO_2_ required to generate 1 MPa after one day can be evaluated. This approach can be used to evaluate the ecological performance at an early age, as in most cases there are no influences on the durability of the concrete at this stage and the strength is therefore the appropriate parameter for evaluating the performance such as for precast elements.

c_i,1d_ = GWP_CO2,e_/*f*_sample,1d_ (4)

**Figure 9:**
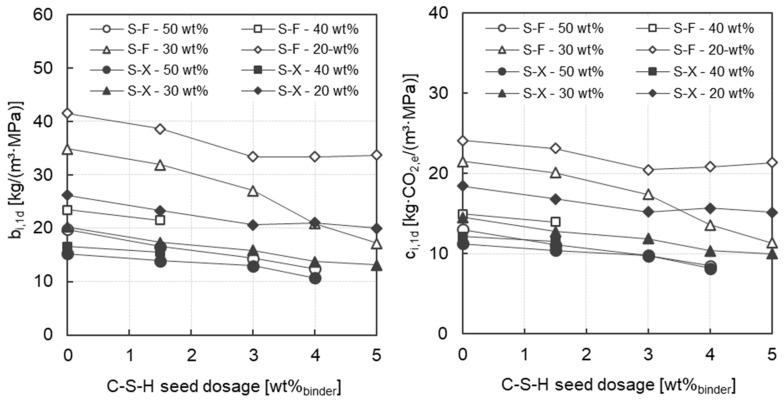
Early age binder intensity b_i,1d_ as a function of the C-S-H seeds dosage (**left**) and early age CO_2_-intensity c_i,1d_ as a function of the C-S-H seeds dosage (**right**) for all mortars investigated.

Figure 9 shows both the early binder intensity b_i,1d_ (left) and the early CO_2_-intensity c_i,1d_ (right) of all mortars investigated as a function of the C-S-H seed dosage. For mortars without C-S-H seeding admixtures, the binder intensity decreases from 41 kg/(m^3^·MPa) to 20 kg/(m^3^·MPa) with increasing clinker content (from 20 wt% to 50 wt%) when using fine slag (S-F). When instead using the very fine slag (S-X), this relationship is shifted to lower values between 26 kg/(m^3^·MPa) and 15 kg/(m^3^·MPa). Due to the higher fineness of the slag, a significantly higher reactivity is already present in this early phase, so that more early strength is developed, and the binder intensity is positively influenced with less clinker. With regard to the effect of C-S-H seeding, it can be seen that the early binder intensity decreases with increasing C-S-H seed dosage regardless of the clinker content and the slag fineness. Especially at a clinker content of 30 wt%, a strong decrease in early binder intensity can be observed at a high dosage of more than 3.0 wt% of C-S-H seeds. In summary, it can be stated that the addition of C-S-H seeds can save around 10 wt% clinker with a comparable early binder intensity.

The early age CO_2_-intensity c_i,1d_ shows comparable correlations with the clinker content, the C-S-H seed dosage and the slag fineness as described above for the early binder intensity b_i,1d_. When using the fine slag (S-F) without C-S-H seeds, the values of c_i,1d_ are in a range of 24 kg·CO_2_/m^3^·MPa and 13 kg·CO_2_/m^3^·MPa. The addition of C-S-H seeds leads to a reduction in c_i,1d_ regardless of the clinker content. However, with a very low clinker content of 20 wt.%, no further decrease can be observed at a dosage of >3.0 wt.% C-S-H seeds. No further increase in early age strength could be achieved with these mortars due to the high dosage of C-S-H seeds (cf. Section 3.2.2). The mortars with 50 wt% clinker and a high C-S-H seed dosage of 4.0 wt% show the lowest c_i,1d_-values of 8.5 kg·CO_2_/m^3^·MPa (S-F) and 8.0 kg·CO_2_/m^3^·MPa (S-X), respectively.

**Figure 10:**
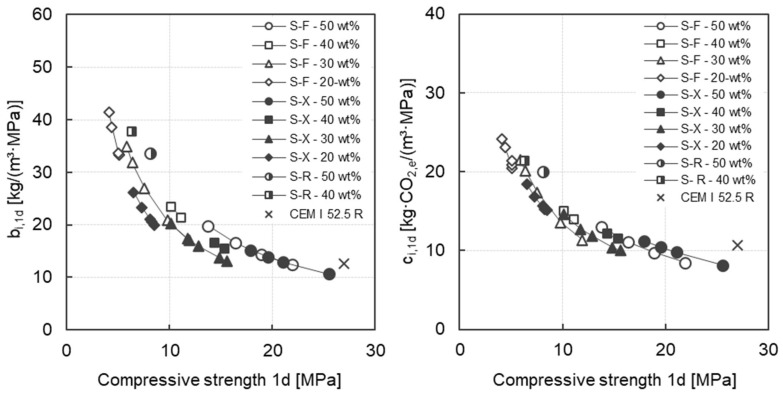
Early age binder intensity b_i,1d_ as a function of the early strength after 1d (**left**) and early age CO_2_-intensity c_i,1d_ as a function of the early strength after 1d (**right**) for all mortars investigated.

Figure 10 presents both the calculated early age binder intensity b_i,1d_ and the early age CO_2_-intensity c_i,1d_ as a function of the early strength (1d) for all mortars investigated. The variability of the data is low. With increasing early strength, a potential decrease in the characteristic values can be observed. Regardless of the clinker content, a decrease in the b_i,1d_-values can be observed with increasing C-S-H seed dosage. From a clinker content of 30 wt%, the seed dosage can compensate for the granulometry effect by the finer slag if the C-S-H dosage is sufficiently high. For example, for a clinker content of 30 wt%, the mortar with fine slag (S-F) and a C-S-H seed dosage of 5.0 wt% and the mortar with very fine slag (S-X) without C-S-H seeds show comparable b_i,1d_-values of 20.8 (S-F) and 20.2 (S-X). The binder in these mortars is therefore consumed similarly in the early phase, regardless of the fineness of the slag. The dosage level of the C-S-H seeds, which leads to an equalization of the b_i,1d_ values, depends on the clinker content. With increasing clinker content, a lower C-S-H dosage is necessary. Only with a very low clinker content of 20 wt% does the addition of C-S-H seeds not lead to a comparable reduction in the b_i,1d_ values. The increase in slag fineness has a stronger effect with these mortars. The mortar with a clinker content of 50 wt% and the very fine slag (S-X) shows a comparable early strength and a comparable b_i,1d_-value to the reference mortar with CEM I 52.5 R.

The observed minimum c_i,1d_-value tends to decrease with increasing early compressive strength up to 15 MPa. Above 15 MPa, the minimum observed c_i,1d_ value appears to reach a plateau in the region of approximately 10 kg·CO_2_/m^3^·MPa. Depending on the required early strength, eco-efficient concretes can be produced with a significantly reduced proportion of clinker with mechanical and chemical activation and therefore a significantly reduced CO_2_ footprint.

## 4. Conclusions

The impact of grinding fineness of slag and hydration accelerators based on C-S-H seeding on the early strength of eco-efficient mortars with low clinker content and ternary blended cements with high contents of slag and limestone powder was investigated comprehensively. The particle size distribution of the slag was varied in two steps. In addition, C-S-H seeds were used for acceleration of the hydration reaction. By combining the two procedures, both methods could be evaluated together.

The granulometric adjustment of the slag enables a significant reduction of the clinker when considering the compressive strength after 28 days. Thus, with increasing fineness of the slag (R → F → X) and a constant clinker content, a denser pore structure and a more efficient microstructure can be assumed. Comparable results were obtained with the raw materials used here and in another study on concretes [29]. Furthermore, it can be summarized that the granulometric adjustment of the slag leads to an improvement in compressive strength especially in the early phase. For example, the clinker content can be reduced from 50 wt% to 30 wt% while maintaining a comparable compressive strength of 10 MPa at 1 day by increasing the slag fineness.

C-S-H seeding enhanced the hydration reaction at early ages of 1 day. However, the strength after 28 days remains unaffected by C-S-H seeding. As the C-S-H dosage level increases, a clear increase in early strength can be observed with a clinker content higher than 30 wt%. With a clinker content of 20 wt%, however, the increase in compressive strength stagnates at a C-S-H dosage level of 3.0 wt%.

The results of this study show that both granulometric approaches as well as admixtures are suitable pathways for the design of eco-efficient mortars with low clinker content and high early strength. In particular, the binder phase is responsible for the strength development of concrete, so these findings can be easily transferred from mortar to concrete. Comparable findings are described in the literature [60]. In addition, C-S-H seeds are already used in practice as admixtures [61], so that they can also be easily applied to concretes with a reduced clinker content. Accordingly, ternary blended cements with high contents of granulated blast furnace slag and limestone powder can also be used for the production of precast concrete elements, considering the granulometric properties and the use of C-S-H seeds, which can lead to a significant reduction in the resulting CO_2_ emissions.

## Figures and Tables

**Table 1:** Physical properties of the binder components.

Binder Component	Density	d_10_	d_50_	d_90_	Specific Surface Area	Initial Setting Time	Water Demand	Compressive Strength (28 d)
	(g/cm^3^)	(μm)	(μm)	(μm)	(cm^2^/g)	(min)	(Vol%)	(MPa)
CEM I 52.5 R	3.106	1.1	8.6	27.4	5800	95	44.5	70.9
Mikrodur R-F slag	3.122	0.8	5.2	12.4	7200	-	51.5	57.2
Mikrodur R-X slag	2.902	0.7	2.6	5.3	10100	-	56.6	74.8
Blast furnace slag	2.906	1.9	12.1	33.9	4400	-	47.3	-
Limestone powder ^1^	2.740	1.7	11.3	91.2	5100	-	32.4	-

^1^ Data according to [45].

**Table 2:** Oxide compositions of the binder components.

Binder Component	CaO	SiO_2_	Al_2_O_3_	Fe_2_O_3_	K_2_O	Na_2_O	SO_3_	LOI
	(wt%)							
CEM I 52.5 R	68.9	16.3	4.5	2.9	0.9	0.2	3.4	3.27
Mikrodur R-F slag	41.4	28.6	10.3	0.5	0.8	0.2	2.3	1.06
Mikrodur R-X slag	45.2	29.5	10.1	0.7	0.8	0.3	2.0	0.84
Blast furnace slag	34.2	24.9	6.2	0.2	0.6	0.3	2.4	0.78
Limestone powder ^1^	52.7	2.5	0.6	0.4	0.1	0.02	0.3	41.81

^1^ Data according to [45].

**Table 3:** Mortar compositions.

Mix ID	Cement	Slag	Mikrodur R-F Slag	Mikrodur R-X Slag	Limestone	Water	Fine Aggregate (0/2)	w/c	w/b
	[kg/m^3^]							[-]	
CEM I 52.5	522	-	-	-	-	235	1511	0.45	0.45
C50-S30-LL20-R	261 ^1^	157	-	-	104			0.90	0.45
C50-S30-LL20-F	242 ^1^		175^2^	-	104			0.90	0.45
C50-S30-LL20-X	242 ^1^		-	175 ^2^	104			0.90	0.45
C40-S30-LL30-R	209 ^1^	157	-	-	157			1.13	0.45
C40-S30-LL30-F	190 ^1^	-	175^2^	-	157			1.13	0.45
C40-S30-LL30-X	190 ^1^	-	-	175 ^2^	157			1.13	0.45
C30-S30-LL40-R	157 ^1^	157	-	-	209			1.50	0.45
C30-S30-LL40-F	138 ^1^	-	175^2^	-	209			1.50	0.45
C30-S30-LL40-X	138 ^1^	-	-	175 ^2^	209			1.50	0.45
C20-S30-LL50-R	104 ^1^	157	-	-	261			2.25	0.45
C20-S30-LL50-F	86 ^1^	-	175^2^	-	261			2.25	0.45
C20-S30-LL50-X	86 ^1^	-	-	175 ^2^	261			2.25	0.45

^1^ Cement = CEM I 52.5 R; ^2^ assumption: average clinker content ≈ 12 wt.% according to DIN EN 197-1 [42] (CEM III/C = 5 to 19 wt.% clinker).

**Table 4:** Mixing regime.

Time	Step Description	Duration
[sec]	[cm^2^/g]	[sec]
0–30	Mixing all dry components—fine aggregate and binder	30
31–60	Addition of water including superplasticizer (C-S-H seeds in individual cases)	30
61–300	Mixing	240

**Table 5:** Ultrasonic pulse velocities v after 180, 360, 720 and 1440 min of hydration, respectively, for different binder component fineness (S-R, S-F, S-X) and clinker contents of 20 wt%, 30 wt% and 40 wt%, respectively.

Mix ID	v_180_	v_360_	v_720_	v_1440_
	[m/s]			
C40-S30-LL30-R	857	1363	1643	2155
C40-S30-LL30-F	1173	1553	2000	2424
C40-S30-LL30-X	1026	1539	2073	2649
C30-S30-LL40-R	814	1349	1584	1926
C30-S30-LL40-F	1389	1601	1869	2254
C30-S30-LL40-X	1097	1555	2005	2477
C20-S30-LL50-R	870	1388	1572	1686
C20-S30-LL50-F	1245	1527	1691	1990
C20-S30-LL50-X	1209	1566	1884	2286

**Table 6:** Ultrasonic pulse velocities at 180, 360, 720 and 1440 min of hydration depending on C-S-H seeding dosage and clinker content of 40 wt% and 30 wt%—all mortars shown are with fine slag.

Mix ID	v_180_	v_360_	v_720_	v_1440_
	[m/s]			
C40-S30-LL30-F	1173	1553	2000	2424
C40-S30-LL30-F-1.5	1285	1606	2010	2477
C40-S30-LL30-F-3.0	1271	1629	2046	2519
C30-S30-LL40-F	1387	1600	1869	2254
C30-S30-LL40-F-1.5	1377	1619	1923	2339
C30-S30-LL40-F-3.0	1400	1650	2025	2402

**Table 7:** Values of the relative increments in compressive strength after 1 day (r_act,C-S-H_) of all mortars investigated (n.d. = not determined).

Clinker Content	Slag Fineness	0.0	1.5	3.0	4.0	5.0
		[-]				
50	S-F	1.00	1.19	1.37	1.59	n.d
40	S-F	1.00	1.09	n.d.	n.d.	n.d.
30	S-F	1.00	1.09	1.29	1.68	2.03
20	S-F	1.00	1.07	1.24	1.24	1.23
50	S-X	1.00	1.09	1.18	1.42	n.d.
40	S-X	1.00	1.07	n.d.	n.d.	n.d.
30	S-X	1.00	1.16	1.27	1.47	1.54
20	S-X	1.00	1.12	1.27	1.25	1.31

**Table 8:** Global warming potential data.

Raw Material	GWP [kg·CO_2_/kg]	Source ^1^
CEM I 52.5 R	0.816	[55]
Mikrodur RF (S-F)	0.296	[56] ^2^
Mikrodur RX (S-X)	0.506	[56] ^2^
Blast furnace slag (S-R)	0.114	[56] ^2^
Limestone powder	0.028	[56]
Water	0.000256	[55]
Superplasticizer	0.944	[55]
C-S-H seeds	0.528	[57]

^1^ In [55], a comprehensive literature review was conducted considering common databases (e.g., ÖKOBAUDAT) for data collection of each raw material. ^2^ + own assumption, detailed description in [30].

## Data Availability

Data will be made available on request.
